# Activation of the endocytosis pathway stratifies subtypes and therapeutic sensitivity in colorectal cancer

**DOI:** 10.21203/rs.3.rs-10164107/v1

**Published:** 2026-07-05

**Authors:** Heinz Lenz, Hiroyuki Arai, Andrew Elliott, Yan Yang, Alex Farrell, Joshua Millstein, Fang-Shu Ou, Federico Innocenti, Jingyuan Wang, Francesca Battaglin, Priya Jayachandran, Sandra Algaze, Shivani Soni, Wu Zhang, Richard M. Goldberg, Michael Hall, Aaron Scott, Jimmy Hwang, Emil Lou, Benjamin Weinberg, John L. Marshall, Sanjay Goel, Joanne Xiu, W. Michael Korn, Alan Venook

**Affiliations:** university of southern California; St. Marianna University School of Medicine; Caris Life Sciences; Keck School of Medicine of USC; Mayo Clinic; Zhongshan hospital Fudan University; Norris Comprehensive Cancer Center, Keck School of Medicine of the University of Southern California; Norris Comprehensive Cancer Center, Keck School of Medicine, University of Southern California; of Southern California/Norris Comprehensive Cancer Center, Keck School of Medicine; University of Minnesota; Rutgers Cancer Institute of New Jersey; Caris Life Sciences

## Abstract

Endocytosis regulates receptor trafficking and signaling, yet its role in colorectal cancer (CRC) remains unclear. We analyzed transcriptomic data from 15,025 CRC tumors to evaluate gene expression in clathrin-mediated endocytosis (CME) and the endosomal sorting complexes required for transport (ESCRT) pathway. Pathway-level signatures were compared across consensus molecular subtypes (CMS) and examined in relation to oncogenic signaling programs, while individual endocytosis-related genes were evaluated for associations with clinical outcomes in an independent randomized trial cohort. Expression of CME and ESCRT pathways varied by CMS, with the highest expression in CMS4 and the lowest in CMS3. Endocytosis signatures correlated with multiple oncogenic signaling pathways, including MAPK, TGF-β, WNT, PI3K, NOTCH, and angiogenesis, suggesting broad links between endocytic activity and tumor biology. In the randomized phase III CALGB/SWOG 80405 trial, low expression of *AP2M1*—a core adaptor in the CME complex—was significantly associated with longer overall survival and progression-free survival in patients treated with anti-EGFR agents, but not in those receiving anti-VEGF agents. These findings suggest that endocytic trafficking may be associated with oncogenic signaling and differential therapeutic benefit in patients with metastatic CRC. The endocytosis pathway reflects biologically relevant heterogeneity in CRC and may help guide therapeutic stratification.

## Introduction

Endocytosis is a fundamental cellular process that regulates the internalization, trafficking, recycling, and degradation of transmembrane proteins, including many key oncogenic receptors such as EGFR, HER2, FGFR, TGF-β, and Notch receptor families^1^. While traditionally viewed as a mechanism for signal attenuation, endocytosis also contributes to the spatial and temporal regulation of signal transduction, and under certain conditions, can even sustain oncogenic signaling through endosomal platforms^2, 3, 4, 5, 6^. In addition, components of the endocytic machinery have been implicated in the regulation of intracellular effectors, such as KRAS, highlighting their broader involvement in cancer-relating signaling networks^7^.

The endocytic system is composed of distinct but interconnected pathways, including the clathrin-mediated endocytosis (CME) pathway, which facilitates selective internalization of membrane receptors and cargo via clathrin-coated vesicles, and the endosomal sorting complexes required for transport (ESCRT) pathway, which mediates cargo sorting within multivesicular bodies for degradation or recycling^8, 9, 10, 11, 12^. Both pathways are essential for receptor trafficking, membrane homeostasis, and intracellular signal control.

Recent proteogenomic studies have shown that endocytosis-related proteins are globally downregulated in colorectal cancer (CRC) cells compared to normal colonic epithelium^13^. However, it remains unclear how this downregulation affects tumor biology, especially given the dual role of endocytosis in both signal attenuation and maintenance. Notably, CRC is a highly heterogeneous disease composed of molecularly defined subtypes with distinct signaling architectures^14^. Yet, it remains unclear how endocytic pathways contribute to intracellular signaling networks across these subtypes, and whether their activity is altered in a uniform or subtype-specific manner.

Here, we conducted a large-scale transcriptomic and genomic analysis of over 15,000 CRC tumors from real-world patients to comprehensively characterize the activity of the endocytosis pathway and its associations with oncogenic signaling and immune-related modulators. We additionally explored the association between the endocytosis pathway activity and clinical outcomes in patients receiving standard first-line treatment in a randomized phase III clinical trial (CALGB/SWOG 80405). Our aim was to define the role of endocytosis in shaping CRC biology and to uncover associated molecular dependencies and generate hypotheses for potential therapeutic targeting.

## Methods

### Study population

This study included two distinct cohorts: a molecular profiling cohort and a clinical trial cohort. The molecular cohort consisted of formalin-fixed, paraffin-embedded (FFPE) tumor specimens collected from patients diagnosed with CRC, which were submitted to Caris Life Sciences (Phoenix, AZ), a laboratory certified under the Clinical Laboratory Improvement Amendments (CLIA). These cases were retrospectively reviewed for molecular characterization. The clinical cohort was derived from participants in the CALGB/SWOG 80405 trial (NCT00265850), a randomized phase III study that compared bevacizumab to cetuximab, each administered in combination with either FOLFOX or FOLFIRI, as first-line treatment for patients with metastatic CRC. CALGB is now part of the Alliance for Clinical Trials in Oncology. In the CALGB/SWOG 80405 trial, a total of 1137 patients with centrally confirmed *KRAS* exon 2 wild-type metastatic CRC were randomized to receive cetuximab- or bevacizumab-based chemotherapy (cetuximab arm: n = 578; bevacizumab arm: n = 559)^15^. For the current study, we analyzed RNA sequencing data from tumor tissue samples available in a subset of these patients. Specifically, 433 patients (cetuximab arm: n = 207; bevacizumab arm: n = 226) had sufficient quality and quantity of tumor RNA for whole-transcriptome sequencing (WTS) and were included in this study.

For the Caris cohort, the retrospective study was conducted under Caris Life Sciences’ Research Data Banking protocol, which was reviewed and granted IRB exemption by the WCG IRB. Patients enrolled in the CALGB/SWOG 80405 clinical trial provided written informed consent for molecular research, and the protocol received IRB approval at each participating institution. All study procedures were conducted in accordance with relevant ethical principles, including the Declaration of Helsinki, the Belmont Report, and the U.S. Common Rule.

### Molecular profiling

In the Caris cohort, molecular characterization was performed on FFPE tumor tissues using both DNA- and RNA-based platforms. DNA was analyzed by next-generation sequencing (NGS) utilizing a 592-gene panel (Caris MI TumorSeek), and RNA underwent WTS. Details of sequencing procedures are provided in the Supplementary Methods. For variant interpretation, only those classified as “pathogenic” or “likely pathogenic” under the American College of Medical Genetics and Genomics (ACMG) guidelines were considered mutations, whereas alterations deemed “benign,” “likely benign,” or “variants of uncertain significance” were treated as wild type. The status of microsatellite instability (MSI) and DNA mismatch repair (MMR) was determined through a multimodal approach incorporating targeted NGS and immunohistochemistry, respectively, and tumors were categorized as either MSI-high (MSI-H)/deficient MMR (dMMR) or microsatellite stable (MSS)/proficient MMR (pMMR). PD-L1 expression was assessed by immunohistochemistry using the SP142 monoclonal antibody (Spring Biosciences). Membranous staining intensity in tumor cells was assessed using a four-tiered scale (0, 1+, 2+, 3+). PD-L1 positivity was defined as ≥ 5% of tumor cells showing moderate (2+) or strong (3+) staining. HER2 immunohistochemistry status was defined as positive if ≥ 10% of tumor cells exhibited strong (3+) staining, equivocal if ≥ 50% of tumor cells exhibited moderate (2+) staining, and negative for all other cases. The Caris consensus molecular subtype (CMS) classifier was developed using RNA sequencing data from the WTS platform (Supplementary Methods).

In the CALGB/SWOG 80405 trial cohort, RNA sequencing was conducted using the Illumina HiSeq 2500 system. Expression data were processed and normalized through the MAPRSeq version 3 computational pipeline^16^.

### Statistical analysis

In the Caris cohort, to assess endocytic activity in CRC tumors, we generated composite gene expression signatures for CME (47 genes) and ESCRT (35 genes) pathways (Figure S1), calculated as a sum of individual gene expression z-scores from TPM-normalized expression values (gene lists provided in Supplementary Methods). These composite scores were used to explore associations with clinical features and key molecular biomarkers. Correlative analyses between CME/ESCRT signatures and oncogenic signaling pathway activity were performed using pathway-specific gene sets (Supplementary Methods). For MAPK signaling, we used the MAPK Pathway Activity Score (MPAS), as previously described^17^. Spearman’s rank correlation coefficient was applied to evaluate associations between oncogenic signaling pathway scores and endocytosis activity. Correlation strength was interpreted as follows: coefficients < 0.2 were considered negligible, 0.2–0.39 weak, 0.4–0.59 moderate, and ≥ 0.6 strong. Parallel analyses were conducted for individual genes within the endocytosis pathways.

In the Caris cohort, the expression levels of *CMTM6*, *CMTM4*, and *HIP1R* were analyzed in relation to immune-related features, including PD-L1 protein expression and MSI/MMR status. These genes were selected based on prior reports indicating their roles in the post-translational regulation of PD-L1. Specifically, CMTM6 and CMTM4 have been shown to stabilize PD-L1 at the cell surface by protecting it from lysosomal degradation, whereas HIP1R facilitates lysosomal targeting of PD-L1, thereby downregulating its expression^18, 19, 20^.

In the CALGB/SWOG 80405 cohort, we investigated the association of 8 selected endocytosis-related genes (*CLTC*, *AP2M1*, *GAK*, *TSG101*, *VPS37A*, *PDCD6IP*, *VPS4A*, and *VPS4B*) with overall survival (OS) and progression-free survival (PFS) in patients receiving cetuximab- or bevacizumab-based first-line therapy. These 8 genes were selected because they represent core components of the endocytosis pathway and their essential regulators, encompassing both CME- (*CLTC*, *AP2M1*, and *GAK*) and ESCRT-mediated trafficking and degradation processes (*TSG101*, *VPS37A*, *PDCD6IP*, *VPS4A*, and *VPS4B*), and because their functional relevance to solid tumors including CRC has been previously reported^21, 22, 23, 24, 25, 26^. OS was defined as the time from trial enrollment to death from any cause, and PFS was defined as the interval until radiologic progression or death. Gene expression levels were dichotomized into high and low groups based on median values. Multivariable Cox proportional hazards models were used to estimate hazard ratios for survival endpoints, adjusting for age, sex, race/ethnicity, ECOG performance status, tumor sidedness, number of metastatic sites, *RAS* and MSI status, and chemotherapy backbone regimen. Analyses were performed separately for each treatment arm.

Patients with missing data for any included variable were excluded from the relevant analyses. In the Caris cohort, where a large number of molecular features were analyzed, *p*-values adjusted using the Benjamini–Hochberg method were calculated to account for multiple testing. In the CALGB/SWOG 80405 cohort, molecular data were limited to a smaller number of selected genes, multiple testing correction was not applied. All statistical tests were two-sided with a significance threshold of 0.05. Analyses were conducted using SPSS version 23 (IBM SPSS Statistics) and R version 4.1.0 (R Foundation for Statistical Computing, Vienna, Austria).

## Results

### Caris cohort: patient characteristics and genomic alterations in endocytosis-related genes

A total of 15,025 CRC samples were included in the molecular profiling cohort derived from the Caris dataset. The median patient age was 62 years. Left-sided tumors accounted for 64.1% of cases, and right-sided tumors for 35.9%. Samples were obtained from the primary tumor site in 56.6% and from metastatic sites in 43.4% (Table S1). No pathogenic or likely pathogenic mutations were detected in the 17 endocytosis-related genes included in the Caris MI TumorSeek panel. Gene amplifications were generally infrequent; the most commonly amplified gene was *ARFRP1* (1.03%, Table S2).

### Subtype-specific profiles in endocytosis pathways

CME pathway activity was examined across subgroups defined by *RAS*/*BRAF* mutation status, MSI/MMR status, tumor sidedness, and CMS. Median CME z-scores were significantly higher in MSS/pMMR tumors compared to MSI-H/dMMR (0.33 vs −5.69, *p* < 0.0001) tumors (Fig. 1A). No significant differences were observed by *RAS*/*BRAF* status or primary tumor location (Fig. 1B-C). Notably, CME activity was highest in CMS4 tumors and lowest in CMS3 (median z-scores: CMS1 = −3.76, CMS2 = −5.81, CMS3 = −16.45, CMS4 = 15.81; *p* < 0.0001) (Fig. 1D). Heatmap analysis showed that nearly all CME genes were upregulated in CMS4 and broadly downregulated in CMS3 (Fig. 1E).

Similar trends were observed for the ESCRT pathway. ESCRT z-scores were significantly higher in MSS/pMMR tumors compared to MSI-H/dMMR (median, 0.18 vs −2.55; *p* < 0.01), with no differences by *RAS*/*BRAF* status or sidedness (Fig. 2A–C). Again, pathway activity was highest in CMS4 and lowest in CMS3 (median z-scores: CMS1 = −2.57, CMS2 = −1.86, CMS3 = −12.85, CMS4 = 9.56; *p* < 0.0001) (Fig. 2D), with heatmaps revealing robust expression of ESCRT genes in CMS4 and reduced expression in CMS3 (Fig. 2E).

### Association between endocytosis and oncogenic signaling pathways

CME and ESCRT pathway activities were strongly correlated (ρ = 0.98, *p* < 0.0001) (Figure S2). CME signature scores were strongly and positively correlated with all six major oncogenic signaling pathways—WNT, MAPK, PI3K/AKT/mTOR, angiogenesis, TGF-β, and NOTCH (ρ = 0.78–0.98, all *p* < 0.0001) (Fig. 3A). ESCRT signatures showed similar positive correlations (ρ = 0.77–0.97, all *p* < 0.0001) (Fig. 3B). Expression levels of *VPS4A* and *VPS4B*, genes encoding core ESCRT components, were also strongly and positively correlated with all six pathway scores. For *VPS4A*, ρ ranged from 0.68 to 0.83 (all *p* < 0.0001), and for *VPS4B*, ρ ranged from 0.65 to 0.82 (all *p* < 0.0001) (Fig. 3C–D).

### Association between endocytosis pathways and HER2 protein expression

CME and ESCRT pathway scores did not significantly differ between HER2–positive and HER2–negative tumors (median CME z-scores: 1.31 vs −4.23, *p* = 0.12; median ESCRT z-scores: 2.66 vs −2.67, *p* = 0.20) (Fig. 4A–B). Likewise, *VPS4A* and *VPS4B* expression levels were not associated with HER2 status (median *VPS4A* z-scores: 4.11 vs 4.00, *p* = 0.35; median *VPS4B* z-scores: 4.19 vs 4.25, *p* = 0.69) (Fig. 4C–D).

### Association between endocytosis-related genes and immune-related markers

*CMTM6* and *CMTM4* expression levels were strongly correlated (ρ = 0.62, *p* < 0.0001) (Figure S3). *CMTM6* expression was elevated in MSI-H/dMMR tumors compared to MSS/pMMR (median z-scores, 37.06 vs 32.68, *p* < 0.0001), and in PD-L1–positive tumors compared to PD-L1–negative tumors (median z-scores, 37.92 vs 32.77, *p* < 0.0001) (Fig. 5A–B). In contrast, *CMTM4* expression was lower in MSI-H/dMMR tumors compared to MSS/pMMR tumors (median z-scores, 7.83 vs 8.38, *p* < 0.0001) and in PD-L1–positive tumors compared to PD-L1–negative tumors (median z-scores, 6.19 vs 8.43, *p* < 0.0001) (Fig. 5D–E). Similarly, *HIP1R* expression was lower in MSI-H/dMMR tumors compared to MSS/pMMR tumors (median z-scores, 11.99 vs 13.95, *p* < 0.0001) and in PD-L1–positive tumors compared to PD-L1–negative tumors (median z-score, 12.60 vs 13.85, *p* < 0.001) (Fig. 5G–H). None of these genes (*CMTM6*, *CMTM4*, *HIP1R*) showed a meaningful correlation with tumor mutational burden (Fig. 5C, 5F, 5I).

### CALGB/SWOG 80405 cohort: association between endocytosis-related genes and clinical outcomes

In the CALGB/SWOG 80405 cohort, 433 patients (207 receiving cetuximab and 226 receiving bevacizumab) were analyzed. Baseline characteristics are shown in Table S3. Among the 8 selected endocytosis-related genes, *AP2M1* expression was significantly associated with clinical outcomes in univariable analysis (Table S4–S5). In the cetuximab-treated group, low *AP2M1* expression was associated with prolonged PFS (median 13.3 vs. 8.9 months, log-rank *p* < 0.01) and OS (median 39.4 vs. 20.9 months, log-rank *p* < 0.01) compared to high expression (Fig. 6A–B). These associations remained significant after multivariable adjustment. No survival difference by *AP2M1* expression was observed in patients receiving bevacizumab (Fig. 6C–D). Significant interactions were observed between *AP2M1* expression and treatment group for both PFS and OS (*p* < 0.01). In contrast, *CLTC* expression was associated with OS only in the multivariable analysis in the cetuximab group, but no significant interaction was observed between *CLTC* expression and treatment arm (Table S4–S5).

To further explore *AP2M1*’s biological relevance, we assessed its expression in the Caris cohort. *AP2M1* expression showed strong positive correlations with all six oncogenic pathway signatures (Figure S4), and these associations persisted in *RAS*/*BRAF* wild-type tumors, particularly with the MAPK pathway (Figure S5).

## Discussion

This study provides novel insights into how endocytosis pathways shape CRC biology by integrating molecular defined subgroups, immune modulation, and therapeutic outcomes. To our knowledge, this is the first comprehensive analysis to link large-scale endocytic gene expression profiles with CRC molecular subtypes, oncogenic signaling, and treatment response, using both a real-world cohort of over 15,000 tumors and an independent randomized phase III clinical trial dataset. Our analysis combining multi-omic molecular data with clinical outcomes suggested that endocytosis pathways dynamically modulate tumor progression and therapeutic susceptibility.

The distinct expression patterns of CME and ESCRT pathway genes across CMS highlight the biological specificity of endocytic activity. CMS4 tumors, which are defined by strong TGF-β signaling, stromal infiltration, and angiogenesis, showed the highest expression of endocytosis signatures^14^. This supports the hypothesis that CMS4 tumors rely on integrative signaling networks requiring robust intracellular trafficking and receptor modulation. In contrast, CMS3 tumors, driven primarily by metabolic reprogramming and often harboring *KRAS* mutations, exhibited the weakest association with endocytosis gene expression, suggesting reduced dependence on receptor-mediated signaling dynamics. This may reflect the biology of *KRAS*-mutant tumors, in which constitutive downstream activation of the MAPK and PI3K pathways can occur independently of receptor trafficking. As a result, CMS3 tumors may rely less on endocytic regulation of cell surface signaling activity.

Expression of genes in the CME and ESCRT pathways showed strong correlations with multiple oncogenic signaling pathways, including MAPK, WNT, TGF-β, and PI3K. These findings are particularly intriguing in light of previous reports that described global downregulation of endocytosis-related proteins in CRC cells compared to normal colonic epithelium, suggesting that the pathway may not play a conventional oncogenic driver role^13^. However, our data reveal that specific components of the endocytic machinery—notably those involved in late-stage trafficking such as VPS4A and VPS4B—are selectively retained or even upregulated in certain tumor subtypes such as CMS4, where they closely track with oncogenic pathway activation. This discrepancy suggests that while global endocytosis may be suppressed, tumor cells may actively preserve or repurpose parts of the machinery to sustain key signaling outputs. In this setting, endocytic regulation may represent a subtype-specific dependency and a potential therapeutic vulnerability.

We also identified *AP2M1*, a core component of the CME complex, as a gene of both functional and clinical relevance. Its expression positively correlated with MAPK pathway activity. Paradoxically, however, tumors with low *AP2M1* expression showed better response to cetuximab. This apparent contradiction reflects the dual role of EGFR endocytosis. AP2M1 is essential for clathrin-coated pit formation and facilitates EGFR internalization^27^. While internalization can downregulate receptors, it may also sustain signaling through receptor recycling and endosomal signaling—mechanisms known to maintain MAPK activity^3^. Thus, high *AP2M1* expression may support EGFR-dependent MAPK signaling from endosomes but reduce EGFR surface availability for antibody binding. Conversely, low *AP2M1* expression could prolong EGFR presence on the membrane, enhancing cetuximab binding despite lower downstream signaling. These findings illustrate a critical disconnect: EGFR signaling activity and therapeutic sensitivity to anti-EGFR antibodies do not necessarily align. *AP2M1* may serve as a biomarker that reflects both EGFR pathway reliance and likelihood of benefit from EGFR-targeted therapy, emphasizing the clinical importance of receptor trafficking dynamics.

Although we explored transcript-level expression of immune-related components such as CMTM6, CMTM4, and HIP1R, which have been implicated in PD-L1 recycling^28^, their associations with PD-L1 expression and MSI status were weak and inconsistent. This indicates that while endocytosis may play a role in shaping the immune microenvironment, its contribution in CRC appears limited and warrants further mechanistic investigation.

Our study has several limitations. The analyses were primarily correlative and based on transcriptomic data. Functional validation, such as protein-level assessments or in vivo modeling, will be critical to confirm mechanistic hypotheses. Moreover, while we observed a predictive association between *AP2M1* expression and cetuximab response, it has not yet been prospectively validated as a biomarker. Further studies in independent and longitudinal cohorts are needed to determine their clinical utility.

Nevertheless, our findings provide a foundational framework for understanding how endocytic pathways contribute to CRC progression, subtype specification, and therapeutic vulnerabilities. They also highlight the need to move beyond canonical genetic alterations and consider intracellular trafficking dynamics in the design of future precision oncology strategies. The preferential upregulation of endocytosis pathway genes in CMS4 tumors may reflect a selective pressure to maintain receptor trafficking machinery in highly proliferative or signaling-intensive environments. In this context, targeting the endocytic machinery—particularly in CMS4 tumors that appear to rely on sustained receptor turnover and intracellular signal maintenance—may represent a promising therapeutic strategy, warranting further investigation.

Together, our findings shed new light on the understanding of the endocytosis pathway in CRC biology. By integrating transcriptomic and clinical outcome data, we uncover functional links between endocytic components and oncogenic signaling activity, as well as therapeutic responsiveness. These insights open new avenues for precision oncology, where the status of intracellular trafficking machinery may inform both prognostic stratification and the rational selection of targeted therapies.

## Data Availability

De-identified datasets analyzed in the current study are available from the corresponding author on reasonable request.
